# Pre‐ and Postnatal Predictors of Chronic Lung Disease in Congenital Diaphragmatic Hernia

**DOI:** 10.1002/ppul.71887

**Published:** 2026-11-10

**Authors:** Johannes Hartmann, Johannes Schmidt, Meike Weis, Svetlana Hetjens, Stefan Schoenberg, Michael Boettcher, Florian Kipfmueller, Thomas Schaible, Alba Perez‐Ortiz

**Affiliations:** ^1^ Department of Neonatology and Pediatric Intensive Care Medicine University Medical Center Mannheim, University Heidelberg Heidelberg Germany; ^2^ Department of Radiology and Nuclear Medicine University Medical Center Mannheim, University Heidelberg Heidelberg Germany; ^3^ Department of Medical Statistics and Biomathematics, Medical Faculty Mannheim University Heidelberg Heidelberg Germany; ^4^ Department of Pediatric Surgery University Medical Center Mannheim, University Heidelberg Heidelberg Germany

**Keywords:** chronic lung disease, congenital diaphragmatic hernia, extracorporeal membrane oxygenation, neonatal lung disease, respiratory failure

## Abstract

**Background:**

Chronic lung disease (CLD) is a major contributor to long‐term morbidity in infants with congenital diaphragmatic hernia (CDH). While prenatal predictors such as fetal lung volume and liver herniation are established, the role of early postnatal and clinical course–related parameters in predicting CLD remains insufficiently defined.

**Objective:**

To identify clinical factors associated with the development of CLD in CDH survivors, focusing on prenatal, early postnatal, and clinical course time points.

**Methods:**

We conducted a retrospective single‐center study including 202 infants with CDH and available fetal magnetic resonance imaging (MRI) data. CLD severity was classified at day of life 56, following established criteria analogous to bronchopulmonary dysplasia.

**Results:**

CLD was diagnosed in 116 of 202 infants (57.4%). Prenatal imaging showed lower observed‐to‐expected fetal lung volume (o/e FLV) and more frequent intrathoracic liver and stomach positions among infants with CLD. Early postnatal data indicated lower birth weight, higher arterial PaCO_2_, and elevated oxygenation indices within the first day of life in the CLD group. Clinical‐course parameters such as ECMO support, larger defect size, nitric oxide therapy, and chylothorax were more frequent among infants with CLD. In stepwise multivariable models, discrimination improved from prenatal to early‐postnatal; the best model—o/e FLV, lowest PaCO_2_ within 24 h, and ECMO support (AUC of 0.946).

**Conclusion:**

Significant differences in prenatal and early postnatal parameters were identified between infants with and without CLD. Multivariable logistic regression models demonstrated high predictive accuracy for CLD. These data may facilitate early risk stratification in CDH.

## 1 Introduction

Congenital diaphragmatic hernia (CDH) is a rare but severe developmental defect with still‐relevant neonatal mortality and high rates of pulmonary morbidity among survivors [1]. Pulmonary morbidity in CDH survivors encompasses a range of long‐term respiratory impairments, including obstructive airway disease, impaired gas exchange, and exercise intolerance [2, 3]. The diagnosis of chronic lung disease (CLD) is often used as a clinical marker to identify infants at increased risk for such long‐term pulmonary complications [3]. CLD is commonly defined based on bronchopulmonary dysplasia (BPD) criteria (described by Bancalari and Jobe) as oxygen requirement or the need for respiratory support at day of life (DOL) 28, with subsequent CLD severity grading at DOL 56 [4, 5]. Most cases of CDH are now diagnosed prenatally, and fetal magnetic resonance imaging (MRI) has become a standard component of perinatal evaluation. Prenatal parameters, particularly fetal lung volume and liver herniation assessed by MRI, have been widely studied in CDH and are linked to poor outcome [6, 7, 8]. A recent multicentre study showed that lower o/e FLV is also linked to adverse long‐term respiratory outcomes, underscoring the value of prenatal imaging beyond survival prediction [9].

Despite advances in neonatal care, outcome variability between centers remains high, emphasizing the need for reliable prognostic markers beyond survival alone. Concurrently, with improving survival rates, interest in long‐term respiratory outcomes, especially CLD, continues to grow. While prenatal parameters have been well studied, the predictive value of postnatal factors, such as the oxygenation index (OI) or the PaCO_2_, remains less clear and has mostly been explored in relation to survival, need for extracorporeal membrane oxygenation (ECMO), or pulmonary hypertension (PH) [10, 11, 12]. Their role in predicting long‐term pulmonary outcomes such as CLD is still insufficiently defined. To date, few studies have systematically examined how parameters across the perinatal timeline relate to the risk of CLD.

We therefore aimed to determine whether a combination of prenatal imaging metrics, early postnatal physiological indicators and selected clinical‐course variables predicts CLD in CDH infants. To address this, we analyzed CLD risk in relation to three clinically relevant time points: prenatal (e.g., o/e FLV, liver position), early postnatal (e.g., markers of respiratory stability), and evolving clinical course (e.g., ECMO support, defect size and ventilation duration). An early risk stratification for developing CLD may enable to offer an early, individualized postnatal care and to improve the respiratory management and outcome in CDH.

## 2 Materials and Methods

### 2.1 Study Design and Population

This study was conducted as a monocentric, retrospective observational cohort study at the University Medical Center Mannheim, Germany. We included all patients diagnosed with CDH who received postnatal care at our institution between 2013 and 2021, and who had undergone prenatal fetal MRI with lung volume measurements. Exclusion criteria encompassed patients who died during their initial postnatal hospital stay and those for whom a diagnosis of CLD at Day 56 could not be ascertained. This study was approved by the local ethics committee of the Medical Faculty Mannheim of the University of Heidelberg (approval no. 2025‐820). Owing to the retrospective design and analysis of de‐identified data, the requirement for informed consent was waived.

### 2.2 CLD‐Definition

CLD was diagnosed based on oxygen dependency (FiO2 > 0.21) at DOL 28. CLD severity was stratified at DOL 56 or discharge (whichever occurred first) into mild (no supplemental oxygen required, FiO_2_ ≤ 0.21), moderate (FiO_2_ 0.22–0.29), or severe (FiO_2_ ≥ 0.30 and/or positive pressure support). For the purpose of analysis, we grouped moderate and severe CLD together as “higher‐grade CLD,” as these infants continued to require clinically relevant respiratory support beyond 56 days of life.

### 2.3 Data Collection

Patient data were retrospectively collected from electronic medical records. Extracted variables included demographic characteristics, pre/perinatal data and laboratory results. Fetal lung volume was expressed as observed‐to‐expected fetal lung volume (o/e FLV), and stomach position was classified as intrathoracic according to the method described by Kitano et al. [13] Each infant had an indwelling arterial catheter. Right radial access was preferred, although some infants were catheterized via the left radial or femoral artery. The first arterial blood gas obtained on our neonatal intensive care unit (NICU) was used for the 1‐h values of PaO_2_, PaCO_2_, and OI. OI was calculated as OI = (MAP × FiO_2_/PaO_2_) × 100; MAP in cmH_2_O. In our institution, both the central venous line and arterial catheter are routinely placed in the delivery room, such that the first NICU blood gas typically reflects the infant's status near the end of the first hour of life. The best PaO_2_, OI, and PaCO_2_ values were defined as the most favorable measurements within the first DOL or prior to ECMO initiation. Conventional mechanical ventilation was the primary mode of respiratory support during postnatal stabilization. High‐frequency oscillatory ventilation (HFOV) was used exclusively as an escalation strategy in infants with insufficient gas exchange despite conventional ventilation and was frequently initiated shortly before ECMO cannulation. The indication for ECMO and the exclusive use of veno‐arterial cannulation with a roller pump system were based on the CDH EURO Consortium Consensus Guideline Update 2015 [14]. Relevant criteria included inability to maintain preductal oxygen saturation > 85% or postductal saturation > 70%, increased PaCO_2_ with respiratory acidosis (pH < 7.15) despite optimized ventilatory management, high ventilatory pressure requirements (PIP > 28 cmH_2_O or MAP > 17 cmH_2_O), inadequate oxygen delivery with metabolic acidosis (lactate ≥ 5 mmol/L and pH < 7.15), refractory systemic hypotension despite fluid and inotropic support, and an OI ≥ 40 for at least 3 h. ECMO initiation was based on the overall cardiopulmonary condition and clinical course. In particular, the OI criterion was not invariably met before ECMO initiation when other indicators of severe refractory cardiorespiratory failure were present. Nitric oxide therapy was initiated at 20 ppm when a clinically relevant pre‐ and postductal saturation difference was detected. Weaning from nitric oxide was performed once a stable FiO_2_ requirement below 0.5 was achieved.

### 2.4 Statistical Analysis

Statistical analyses were performed using SAS software (Version 9.4, SAS Institute, Cary, NC, USA). Continuous variables were compared using Student's *t*‐test for normally distributed data and the Wilcoxon two‐sample test for skewed data. Categorical variables were analyzed using chi‐square tests. Stepwise logistic regression analysis was conducted to determine independent predictors for CLD development. Due to its inherent overlap with CLD classification, mechanical ventilation duration was considered conceptually dependent and was therefore excluded from the final predictive models. An exploratory model including ventilation duration was constructed for comparison. Statistical significance was set at a *p*‐value < 0.05.

## 3 Results

### 3.1 Study Population

A total of 545 patients with CDH received postnatal care at our institution between 2013 and 2021. Of these, 92 patients were born at external institutions or had no prenatal diagnosis, and 169 lacked fetal MRI data. These 261 patients were excluded from analysis, resulting in an eligible cohort of 284 patients. Of these, 73 patients died during their initial hospitalization, with 71 of them dying before CLD classification at DOL 56. The final study population included 202 patients who met all inclusion criteria and were evaluated for the presence and severity of CLD at DOL 56. Among these, CLD was diagnosed in 116 cases (57.4%) (Figure 1).

**Figure 1:**
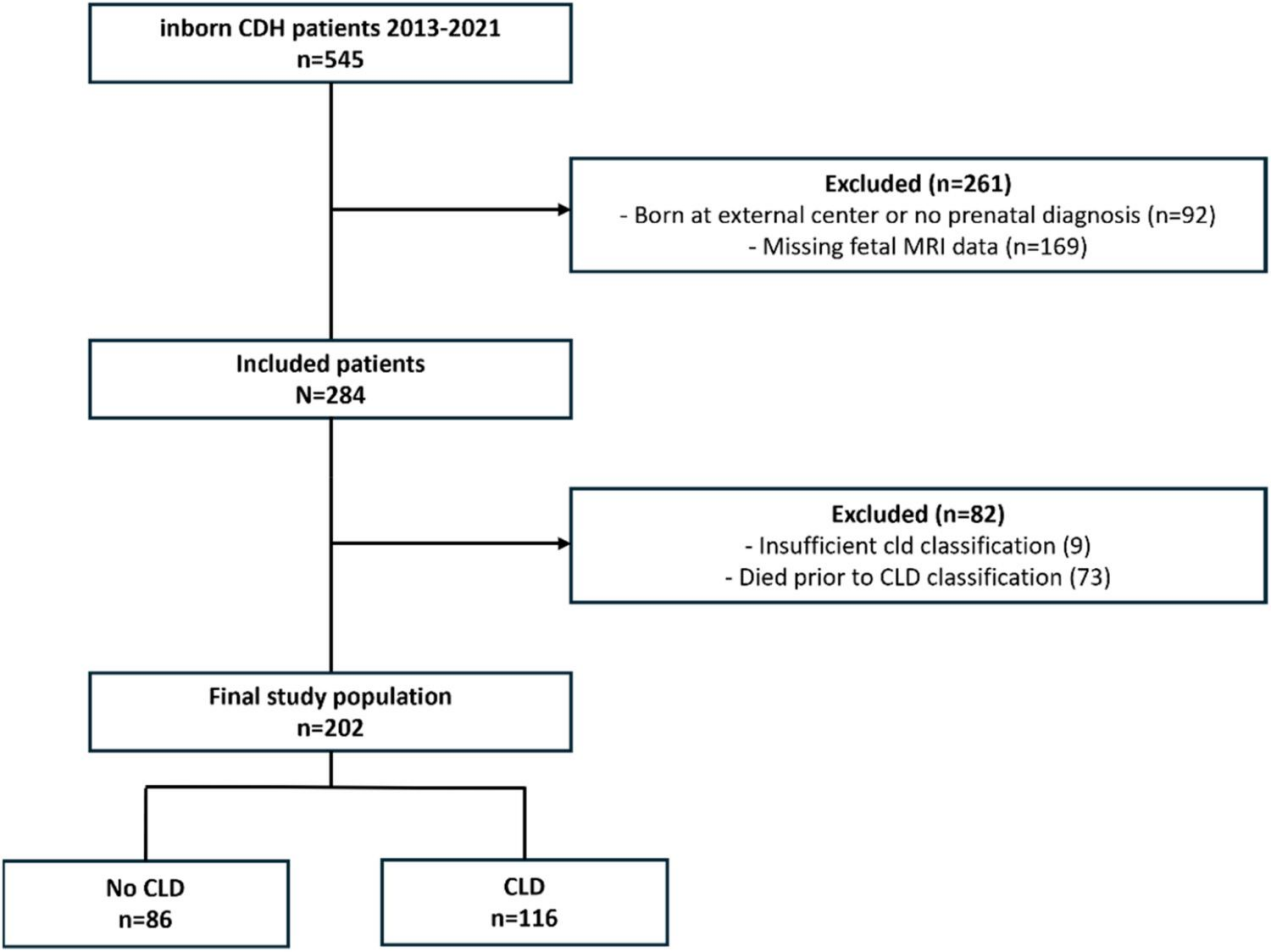
Study flowchart of patient inclusion.

### 3.2 Patient Characteristics

Pre‐ and postnatal clinical characteristics and treatment data in CLD and non‐CLD patients are presented in Table 1. Significant prenatal differences were observed between patients with and without CLD. Right‐sided diaphragmatic defects were more frequent among patients with CLD compared to those without (19.8% vs. 7.0%, *p* = 0.01). Median o/e FLV was significantly lower in the CLD group (27% [IQR: 21–34]) than in the non‐CLD group (37% [IQR: 32–43]; *p* < 0.001). Liver herniation was also more common (84.5% vs. 68.6%; *p* = 0.009) in CLD patients. Patients with CLD had lower birth weight (median: 2988 g vs. 3290 g; *p* = 0.007) and lower gestational age (median: 37.67 weeks vs. 38.07 weeks; *p* < 0.001). ECMO was required significantly more often in the CLD group (36.2% vs. 10.5%; *p* < 0.001). During the first DOL, 43 of 202 infants (21.3%) received HFOV. Of these, 36 (83.7%) subsequently required ECMO support.

**Table 1:** Baseline characteristics and clinical parameters in CDH patients with and without chronic lung disease (CLD). Values are presented as numbers (percentages) or means ± standard deviations, unless otherwise stated. ^1^Stomach position defined after Kitano et al. [13].

Parameter	CLD+	No CLD	*p*‐value
Total patients (%)	116 (57.43%)	86 (42.57%)	
Prenatal characteristics			
Defect site			
− left (%)	93 (80.17)	80 (93.02)	0.01
− right (%)	23 (19.83)	6 (6.98)	
RFLV (MRI) % (SD)	29.42 (± 10.42)	45.57 (± 17.42)	< 0.0001
Lung‐to‐head ratio ultrasound (SD)	38.34 (± 12.41)	47.21 (± 11.66)	< 0.0001
Liver position (intrathoracic)			
− yes (%)	90 (77.59%)	31 (36.05%)	< 0.0001
− no (%)	31 (36.05%)	55 (63.95%)	< 0.0001
Stomach position (MRI)			
0—Abdominal (%)	27 (23.28%)	29 (33.72%)	0.10
1— Partly thoracic (%)	27 (23.28%)	34 (39.53%)	0.013
2— Mostly thoracic (%)	42 (36.21%)	20 (23.26%)	0.047
3— Completely thoracic (%)	20 (17.24%)	3 (3.49%)	0.0023
Postnatal clinical status			
Gestational age (weeks) (SD)	37.67 (± 1.35)	38.07 (± 1.04)	0.0190
Birth weight (g) (SD)	2955 (± 483)	3128 (± 436)	0.0094
PaCO_2_ at 1 h (mmHg) (SD)	48 (± 16)	34 (± 14)	< 0.0001
	*n* = 49	*n* = 36	
Lowest PaCO_2_ 24 h (mmHg) (SD)	51 (± 17)	26 (± 5)	< 0.0001
	*n* = 116	*n* = 80	
Oxygenation index at 1 h (SD)	16.5 (± 11.1)	6.5 (± 6.8)	< 0.0001
	*n* = 113	*n* = 77	
Lowest oxygenation index 24 h (SD)	10.77 (± 9.7)	3.15 (± 2.1)	< 0.0001
	*n* = 116	*n* = 79	
Clinical course and interventions			
Chylothorax (%)	29 (28.71%)	5 (6.17%)	0.0001
Nitric oxide application (%)	94 (89.52%)	24 (29.27%)	< 0.0001
Nitric oxide application (days) (SD)	20.9 ± 12.0	8.2 ± 6.8	< 0.0001
ECMO (%)	76 (65.52%)	4 (4.65%)	< 0.0001
ECMO days (SD)	9.5 ± 3.3	7.2 ± 2.8	0.1704
Defect size			
− A (%)	1 (0.95%)	14 (20.29%)	< 0.0001
− B (%)	21 (20.00%)	39 (56.52%)	< 0.0001
− C (%)	60 (57.14%)	16 (23.19%)	< 0.0001
− D (%)	23 (21.90%)	0	< 0.0001
Ventilation days (SD)	32.82 (± 12.74)	12.43 (± 5.92)	< 0.0001

Abbreviations: A–D, defect size categories after CDH study group staging; CLD, chronic lung disease; ECMO, extracorporeal membrane oxygenation; IQR, interquartile range; MRI, magnetic resonance imaging; NO, nitric oxide; OI, oxygenation index; o/e FLV, observed‐to‐expected fetal lung volume; PaCO_2_, partial pressure of carbon dioxide; SD, standard deviation.

### 3.3 Subgroup Analysis: No CLD vs. Higher‐Grade CLD

A subgroup analysis compared patients without CLD to those with higher‐grade CLD (CLD Grades 2 and 3). Patients in the higher‐grade CLD group had significantly lower prenatal o/e FLV values (median: 26.4% vs. 45.6%; *p* < 0.0001) and lower birth weight (2893 g vs. 3129 g; *p* = 0.011) compared to non‐CLD patients. Patients with higher‐grade CLD required longer durations of mechanical ventilation (48 vs. 12 days; *p* < 0.0001), received more days of inhaled nitric oxide therapy (33 vs. 8 days; *p* < 0.0001), and were more frequently supported with ECMO (90% vs. 4.7%; *p* < 0.0001), with a longer but not significantly ECMO duration (11.0 vs. 7.2 days; *p* = 0.063). Furthermore, parameters of early respiratory function showed significant differences: the OI approximately 1 h after birth was markedly elevated in the higher‐grade CLD group (median: 25.7 vs. 5.4; *p* < 0.0001), as was the lowest OI during the first day or prior to ECMO initiation (14.8 vs. 2.6; *p* < 0.0001). The lowest arterial partial pressure of carbon dioxide (PaCO_2_) was also higher compared to the non‐CLD patients (median: 55.0 vs. 29.4 mmHg; *p* = 0.0011) and at the lowest value recorded during the first day or prior to ECMO (52.2 vs. 26.0 mmHg; *p* < 0.0001; Figure 2).

**Figure 2:**
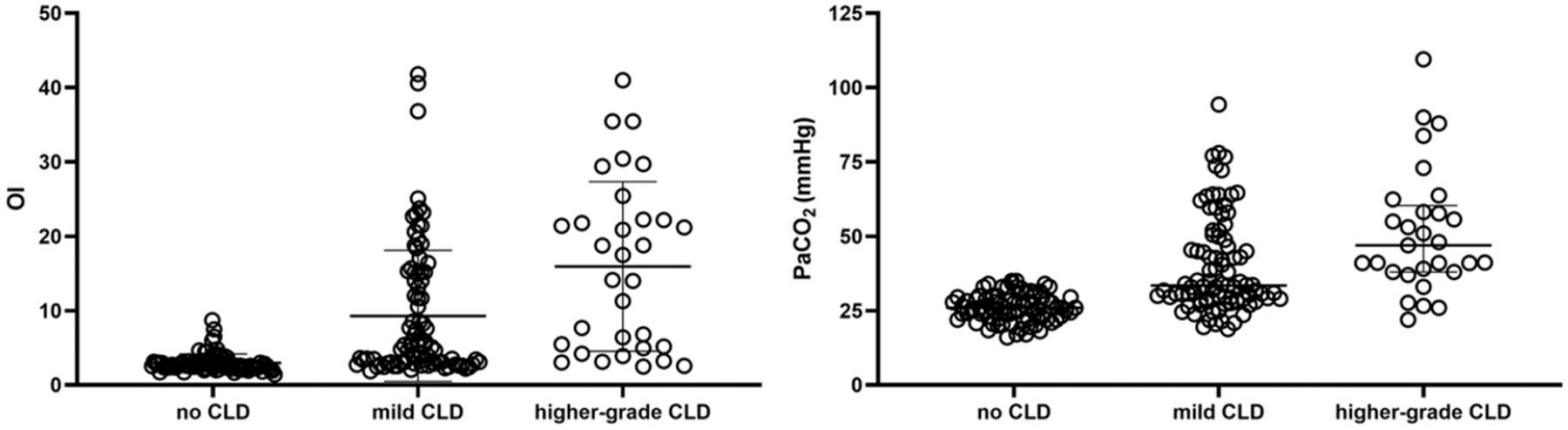
Lowest values of OI (oxygenation index) (left) and PaCO_2_ (right) recorded within the first 24 h of life or before ECMO initiation.

### 3.4 Exploratory Regression Model Including Ventilation Duration

An initial stepwise logistic regression analysis identified prenatal lung volume (o/e FLV) and duration of mechanical ventilation as the two most predictive variables for the development of CLD (OR per 1% o/e FLV: 0.93, 95% CI: 0.87–0.99; OR per day ventilation: 1.27, 95% CI: 1.14–1.41).

### 3.5 Prenatal Prediction Model

A separate logistic regression model based solely on prenatal parameters identified o/e FLV and liver herniation as significant predictors of CLD (OR per 1% o/e FLV: 0.92, 95% CI: 0.89–0.95; OR for liver position: 3.44, 95% CI: 1.71–6.94). The model achieved an AUC of 0.844. A cutoff for o/e FLV around 34% showed a sensitivity of 75% and specificity of 79%.

### 3.6 Early Postnatal Prediction Model (First DOL)

A logistic regression model including both prenatal and early postnatal parameters—specifically o/e FLV (OR per 1%: 0.95, 95% CI: 0.92–0.99), birth weight (OR per 100 g: 0.90, 95% CI: 0.82–0.99), PaCO_2_ (OR per mmHg: 1.19, 95% CI: 1.09–1.29), and OI at 1 h (OR per point: 1.09, 95% CI: 1.04–1.16)—showed improved predictive accuracy for CLD. The model achieved an AUC of 0.909. Cutoff values included o/e FLV < 34%, PaCO_2_ > 35 mmHg, OI > 5.4, and birth weight < 2904 g, with individual sensitivities and specificities ranging between 53% and 79%.

### 3.7 Extended Prediction Model Including Clinical Course (Excluding Ventilation Duration)

A logistic regression model incorporating prenatal, early postnatal, and selected clinical course parameters (see Table 1; ventilation duration excluded) yielded an AUC of 0.946. Variables retained in the final model were o/e FLV (OR per 1%: 0.89, 95% CI: 0.83–0.95), the lowest PaCO_2_ within the first day or prior to ECMO (OR per mmHg: 1.16, 95% CI: 1.03–1.31), and ECMO therapy (reference category: ECMO = yes; OR: 0.19, 95% CI: 0.04–0.87). A cutoff of 33.8% for o/e FLV yielded a sensitivity of 75.9% and specificity of 79.1%; for PaCO_2_, a threshold of 35.2 mmHg resulted in 53.4% sensitivity and 98.8% specificity (Figure 3).

**Figure 3:**
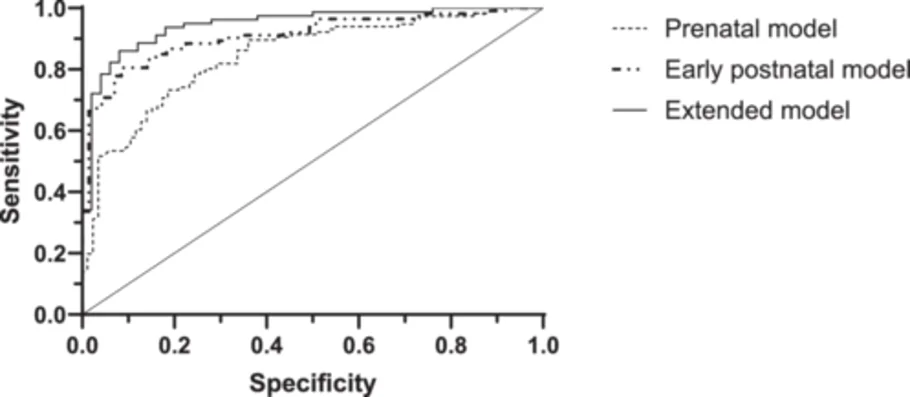
Receiver operating characteristic (ROC) curves for three stepwise logistic regression models predicting chronic lung disease (CLD). Prenatal model: o/e FLV and liver position (AUC = 0.844), Early postnatal model: o/e FLV, birth weight, oxygenation index at 1 h, and arterial PaCO_2_ on the first day (AUC = 0.909), Extended model: o/e FLV, lowest PaCO_2_ within the first day or before ECMO, and ECMO requirement (AUC = 0.946).

### 3.8 ECMO Subgroup Analysis

In the subgroup of patients treated with ECMO (*n* = 80), patients with higher‐grade CLD had significantly longer durations of ECMO therapy (median: 11.0 vs. 6.0 days; *p* = 0.0066) and mechanical ventilation (median: 50.0 vs. 31.0 days; *p* < 0.0001) compared to those with no or mild CLD. No significant differences were observed in prenatal parameters such as o/e FLV within the ECMO subgroup.

Separate logistic regression analyses were conducted. No model using only prenatal parameters or combinations of prenatal and first DOL variables resulted in significant predictive value. A model including all available parameters and ventilation duration yielded an AUC of 0.915. In this model, mechanical ventilation duration was independently associated with moderate to severe CLD (OR per day: 1.20, 95% CI: 1.09–1.32). When ventilation duration was excluded, only ECMO duration remained in the model, with a reduced AUC of 0.715. ECMO duration was likewise independently associated with CLD severity (OR per day: 1.31, 95% CI: 1.09–1.59). A cutoff of 7.39 days for ECMO duration was associated with a sensitivity of 92% and a specificity of 37% for predicting moderate to severe CLD.

## 4 Discussion

CLD remains a major contributor to long‐term morbidity in infants diagnosed with CDH. Our study identified a combination of prenatal, early postnatal, and clinical course parameters that independently predicted the development of higher‐grade CLD in this population. The predictive performance of our logistic regression models improved stepwise with the integration of additional time points from an AUC of 0.844 (prenatal parameters only), to 0.909 (with early postnatal variables), and finally to 0.946 when selected clinical course markers were added. These stepwise improvements reflect the increasing predictive value of integrating data across the perinatal timeline. Our rates of no or mild CLD were comparable to previous studies assessing oxygen requirement at Day 28, while other reports were based on assessment on Day 30 or Day 56, which might limit direct comparability [4, 15, 16].

We identified several significant differences between patients with and without CLD. Prenatal predictors such as o/e fetal lung volume, liver position, and defect size have been well established in the literature as indicators of postnatal severity and survival in CDH [6, 7, 17]. Our findings confirm their relevance, with significantly lower o/e FLV values and higher rates of liver herniation observed among infants who developed CLD. Intrathoracic stomach position was also significantly more frequent in this group, consistent with findings from few earlier studies [18, 19]. In a logistic regression model based solely on prenatal variables, o/e FLV and liver position were independently associated with the development of CLD. Although predictive, their combined discriminatory power remained moderate, indicating that prenatal imaging alone may not sufficiently identify infants at highest risk for CLD.

In addition to prenatal markers, we identified several early postnatal clinical parameters that were independently associated with higher‐grade CLD. Specifically, lower birth weight, elevated OI at 1 h of life, and higher arterial PaCO_2_ levels within the first 24 h demonstrated significant predictive value in our multivariable analysis. These variables were all available within the immediate postnatal period, allowing for early risk stratification. A model incorporating these parameters alongside o/e FLV achieved high discriminative accuracy (AUC = 0.90), underlining the clinical utility of integrating physiological stability measures observed within the first hours of life. Ruttenstock et al. [12] demonstrated that the best OI within the first 24 h is associated with survival to Day 28. Interestingly, our analysis identified the OI at 1 h of life as a stronger predictor of CLD than the best OI in the first 24 h in logistic regression. These findings support the concept of the “Golden Hour,” emphasizing that early cardiorespiratory status may be crucial in determining long‐term pulmonary outcomes [20]. In our cohort, all infants without CLD achieved an OI < 10 and a PaCO_2_ ≤ 35 mmHg during the first DOL. Taken together, the identified thresholds for o/e FLV, PaCO_2_, and OI may provide additional information for early risk stratification when interpreted within the overall clinical context. However, these cohort‐derived cutoffs should be considered exploratory risk markers rather than treatment thresholds and require external validation. However, it is important to emphasize that a PaCO_2_ threshold ≤ 35 mmHg during the first DOL does not reflect a general physiologic recommendation. Rather, this finding must be interpreted in the context of our center's respiratory management strategy, where permissive hypercapnia is explicitly avoided during early postnatal stabilization. In clinical practice, we target a PaCO_2_ range of approximately 30–40 mmHg, adjusted according to arterial pH values. The interpretation of early PaCO_2_ and OI should also take the ventilatory mode into account. At our center, HFOV was not routinely used as an alternative primary ventilation strategy but exclusively represented an escalation of respiratory support. Accordingly, 36 of 43 infants (83.7%) receiving HFOV during the first DOL subsequently required ECMO. HFOV exposure in this cohort should therefore primarily be regarded as a marker of severe early respiratory compromise. Due to the retrospective study design, the ventilatory mode at the exact time of blood gas sampling could not be reliably reconstructed for all patients, which limits the uniform physiological interpretation of these parameters. Early postnatal markers may provide more actionable insights than prenatal metrics alone, which reflect anatomical risk but not functional adaptation. Together, these findings highlight the additive value of early postnatal markers alongside established prenatal parameters for individualized risk prediction.

Looking beyond the immediate postnatal period, several clinical course–related variables showed strong associations with CLD development. Defect size and the presence of chylothorax were more frequent among patients with CLD, confirming previous observations regarding their association with disease severity [21, 22, 23]. Among all parameters, prolonged mechanical ventilation beyond 35 days emerged as the most robust predictor for higher‐grade CLD, consistent with previous studies [16, 24]. However, as ventilation duration is both a consequence and a defining criterion of CLD, it was excluded from the final predictive models to avoid conceptual overlap. The resulting model, which incorporated o/e FLV (prenatal), the lowest PaCO_2_ within the first DOL (early postnatal), and the need for ECMO therapy (clinical course), achieved excellent discriminative performance (AUC = 0.946). Importantly, all three variables are typically available within the first days of life. Although ECMO requirement reflects evolving disease severity and is influenced by center‐specific treatment preferences, it does not by itself fully characterize subsequent pulmonary morbidity. The combination of prenatal anatomical severity, early postnatal physiological adaptation, and the need for ECMO may therefore provide complementary information for early risk stratification.

In our cohort, ECMO was initiated based on a combination of physiological indicators, although predefined thresholds were not uniformly met [14]. In particular, the OI criterion of ≥ 40 for at least 3 h was not invariably met before ECMO initiation when other indicators of severe cardiorespiratory failure were present. This is consistent with ELSO registry data reported by Guner et al. [25], in which approximately one‐third of CDH neonates with available pre‐ECMO OI data had an OI < 40 before ECMO initiation. While ECMO itself was retained as an independent predictor of CLD in our final model, it likely reflects underlying disease severity rather than directly contributing to CLD. Cardiocirculatory factors not included in the present model may contribute to this association. In particular, left ventricular hypoplasia or dysfunction and the severity of PH may influence early gas exchange, ventilatory requirements, and hemodynamic stability and may consequently affect the indication for ECMO [26]. Thus, ECMO may partly serve as a surrogate for cardiopulmonary disease severity that is not captured by prenatal lung metrics or respiratory parameters alone. Given that two‐thirds of ECMO‐treated infants did not develop higher‐grade CLD, the timing and context of ECMO initiation may still play a role [27]. In selected subgroups—particularly infants who are initially stable but later deteriorate (“honeymoon” pattern)—earlier ECMO initiation could limit cumulative lung injury. However, our data were not designed to address this question directly, and ECMO might serve as a composite surrogate for multiple clinical variables such as OI, PaCO_2_, and liver herniation. Further studies are needed to disentangle these associations. Nevertheless, the incremental clinical benefit of the combined model beyond its statistical discrimination requires external validation, particularly given the center‐specific nature of ECMO initiation.

Despite these novel insights, our study has some limitations that must be acknowledged. Several patient groups were excluded (see Figure 1), including infants without prenatal diagnosis or MRI, as well as those who died before CLD classification. This introduces a survivor bias, as the prediction models are restricted to infants who survived long enough for CLD classification and therefore do not reflect the full spectrum of disease severity in the overall CDH population. While these exclusions reduced the sample size, they ensured a homogeneously characterized cohort with standardized imaging‐based risk stratification. Over the past decade, the proportion of patients undergoing fetal MRI has steadily increased at our center, as this modality became more integrated into routine care. In earlier years, prenatal assessment was primarily based on detailed ultrasound. The exclusion of infants without fetal MRI may therefore have introduced selection bias and may limit the representativeness of the study cohort. Perrone et al. [28] demonstrated in a large cohort that MRI‐derived volumetric parameters are predictive of neonatal outcomes, underlining the value of standardized prenatal imaging in CDH. Future studies should aim to include detailed information on comorbidities such as PH and cardiac dysfunction, particularly in infants with initially favorable prenatal parameters [29]. The absence of longitudinal follow‐up data regarding respiratory status after discharge further limits interpretation of long‐term outcomes. Moreover, institutional practices—such as our center's tendency toward early ECMO initiation—may influence both outcomes and generalizability, given considerable variability in clinical strategies across centers.

Clinically, the identification of precise prenatal and perinatal risk factors for higher‐grade CLD holds considerable significance. Our study suggests the potential to enhance early risk stratification, enabling clinicians to identify high‐risk infants promptly. Early identification of these at‐risk patients could allow for individualized management strategies, potentially reducing the severity of CLD and improving overall outcomes. Future prospective studies should focus on validating these novel predictors, exploring the impact of tailored respiratory management strategies, and including robust longitudinal follow‐up to confirm their utility in clinical practice.

## 5 Conclusion

In conclusion, our study identified significant prenatal, peripartal, and early postnatal parameters associated with CLD in infants with CDH. Highlighting the relevance of these parameters contributes to improved risk stratification and emphasizes the importance of incorporating both prenatal and early‐life clinical factors in clinical decision‐making. Future research should validate these findings and explore their implications for personalized patient management and improved clinical outcomes.

## Author Contributions

**Johannes Hartmann:** writing – original draft, conceptualization, methodology, data curation, investigation, formal analysis. **Johannes Schmidt:** conceptualization, methodology, writing – review and editing. **Meike Weis:** methodology, formal analysis, data curation, writing – review and editing. **Svetlana Hetjens:** formal analysis, supervision, writing – review and editing. **Stefan Schoenberg:** writing – review and editing, project administration. **Michael Boettcher:** writing – review and editing, project administration, resources. **Florian Kipfmueller:** writing – review and editing, supervision, resources, project administration. **Thomas Schaible:** writing – review and editing, project administration, resources, supervision. **Alba Perez‐Ortiz:** writing – original draft, project administration, supervision, validation.

## Funding

The authors have nothing to report.

## Conflicts of Interest

The authors declare no conflicts of interest.

## Data Availability

The data that support the findings of this study are available on request from the corresponding author. The data are not publicly available due to privacy or ethical restrictions.
